# Arsenic Species in *Cordyceps sinensis* and Its Potential Health Risks

**DOI:** 10.3389/fphar.2019.01471

**Published:** 2019-12-06

**Authors:** Yaolei Li, Yue Liu, Xiao Han, Hongyu Jin, Shuangcheng Ma

**Affiliations:** ^1^National Institutes for Food and Drug Control, Beijing, China; ^2^School of Chinese Pharmacy, Beijing University of Chinese Medicine, Beijing, China; ^3^Department of Pharmacy, Beihua University, Jilin, China

**Keywords:** *Cordyceps sinensis*, arsenic species, inorganic arsenic, SEC-HPLC-ICP-MS, human health risk assessment

## Abstract

High arsenic residues make *Cordyceps sinensis* a concern in China. Arsenic toxicity is related to its species. Many studies have evaluated the toxicity of total arsenic, but few have studied its species. In this study, the species of arsenic in *C. sinensis* and its potential health risk were investigated. SEC-HPLC-ICP-MS was used to analysis of arsenic in *C. sinensis* and unknown arsenic (uAs) was discovered. Additionally, arsenic in *C. sinensis* was mainly found in alkali-soluble proteins. The trend of arsenic transformation indicated that unknown arsenic in *C. sinensis* may be converted into free inorganic arsenic, which enhanced toxicity. The result of risk assessment indicated that there were potential health risks of uAs. Hereon, we proposed recommendations for the use of *C. sinensis* and regulatory recommendations for arsenic standards. This study contributed to the toxicity reveal, safety evaluation, and risk assessment of arsenic in *C. sinensis*.

## Introduction

*Cordyceps sinensis*, which distributes mainly in alpine regions in Qinghai, Tibet, Sichuan, Yunnan, Gansu, and Guizhou of China (Li et al., 2006; Yang et al., 2009), is a traditional Chinese medicine (TCM) with a long history. It is parasitic in the larvae of the bat moth, making the larvae body rigid, and forms a rod-shaped sub-seat in the head of the worm in summer (Yue et al., 2008; Huang et al., 2018). It’s expensive due to its unique growth environment (Dawn et al., 2012) and important medical value. Compendium of materia medica records its role of protecting the lungs and kidneys, stopping bleeding and removing blood stasis (Li and Tsim, 2004). Annals of Sichuan also records that it works the same as ginseng (Kang et al., 2013). It is included in the 2015 edition of the Chinese Pharmacopoeia (Chinese Pharmacopoeia Committee, 2015).

High arsenic residues and exorbitant price make *C. sinensis* a concern in China. In 2016, the National Medical Products Administration (NMPA) issued that the total arsenic (tAs) was founded to be 4.4–9.9 mg kg^-1^ in *C. sinensis* and its related products. Meanwhile, the consumption indicated that the long-term use may cause health risks (National Medical Products Administration, 2016). This has caused widespread concern of social media and consumers. They have no consensus about arsenic in *C. sinensis*. Some view tAs accumulates in human body and is harmful (Lu et al., 2017), however, other view arsenic is harmless because its level in *C. sinensis* is negligible (China Science Daily, 2016). Thus, it is necessary to explore whether arsenic in *C. sinensis* is safe to humans.

The toxicity of arsenic is closely related to its species (Liu Q et al., 2018), different species have different toxicities. Combined with its chemical forms and toxicological characteristics, arsenic is mainly divided into inorganic arsenic (iAs) and organic arsenic (oAs). Inorganic arsenic has been listed as Group 1 carcinogen by International Agency for Research into Cancer (IARC) (Sharma and Sohn, 2009). The LD_50_ values of iAs including As(III) and As(V) in rat are 14 and 20 mg kg^-1^, respectively (Moe et al., 2016). Organic arsenic such as arsenocholine and arsenosugar are considered to be nontoxic (Liu et al., 2013). The evaluation of arsenic with tAs in TCM has many shortcomings (Zhu et al., 2013). For instance, most of the marine drugs mainly contain arsenosugar, and it’s unreasonable to evaluate the toxicity of arsenic by tAs. Thus, it is necessary to accurately study the arsenic species in *C. sinensis*.

Currently, the Chinese Pharmacopoeia (edition 2015) contains detection methods for six arsenic species (Chinese Pharmacopoeia Committee, 2015). Other national standards such as USP, EP, etc. do not include. There are many research methods and progress (Zhao et al., 2006; Monika and Danuta, 2016; Werner et al., 2018), and most of them concern the detection of small-molecule arsenic compounds in TCM, however only few studies on macromolecular compounds such as arsenosugar, arsenic-binding protein, and other unknown arsenic species. Many studies have evaluated the toxicity of tAs, but few have studied its species (Moreda-Piñeiro et al., 2011; Cao et al., 2015; Zhou et al., 2017; Lu et al., 2017; Sun et al., 2017). Before this work, we had focused on establishing a method to analysis arsenic speciation and test 34 batches of *C. sinensis* using 10% nitric acid (v/v) combined with microwave extraction. This condition was intense enough to make all species convert to iAs and maximized the health risks of arsenic in *C. sinensis*. The results showed that there were only iAs in *C. sinensis*, and As(III) was more abundant than As(V) (Zuo et al., 2018). However, according to the past research, most of the arsenic in biomimetic extraction was not detected (Cao et al., 2015). The administration of *C. sinensis* involves consumption after boiling and cooling, with almost all of it taken into the human digestive system. Hence, considering that it may cause human health and safety risks, it’s very meaningful to study arsenic speciation in *C. sinensis*.

In this study, we first extracted the arsenic species in *C. sinensis* by simulating gastric juice and nitric acid with different proportions. The transformation trend of arsenic species in *C. sinensis* was predicted and the distribution of arsenic was investigated. We discovered the unknown arsenic species by establishing SEC-HPLC-ICP-MS. Furthermore, we conducted a health risk assessment to describe its potential health risks. Finally, the medicinal and regulatory recommendations for *C. sinensis* were provided. Our study provides a valuable guide to the toxicological risk of arsenic in *C. sinensis* for consumers.

## Materials and Methods

### Sample Collection

**Figure 1:**
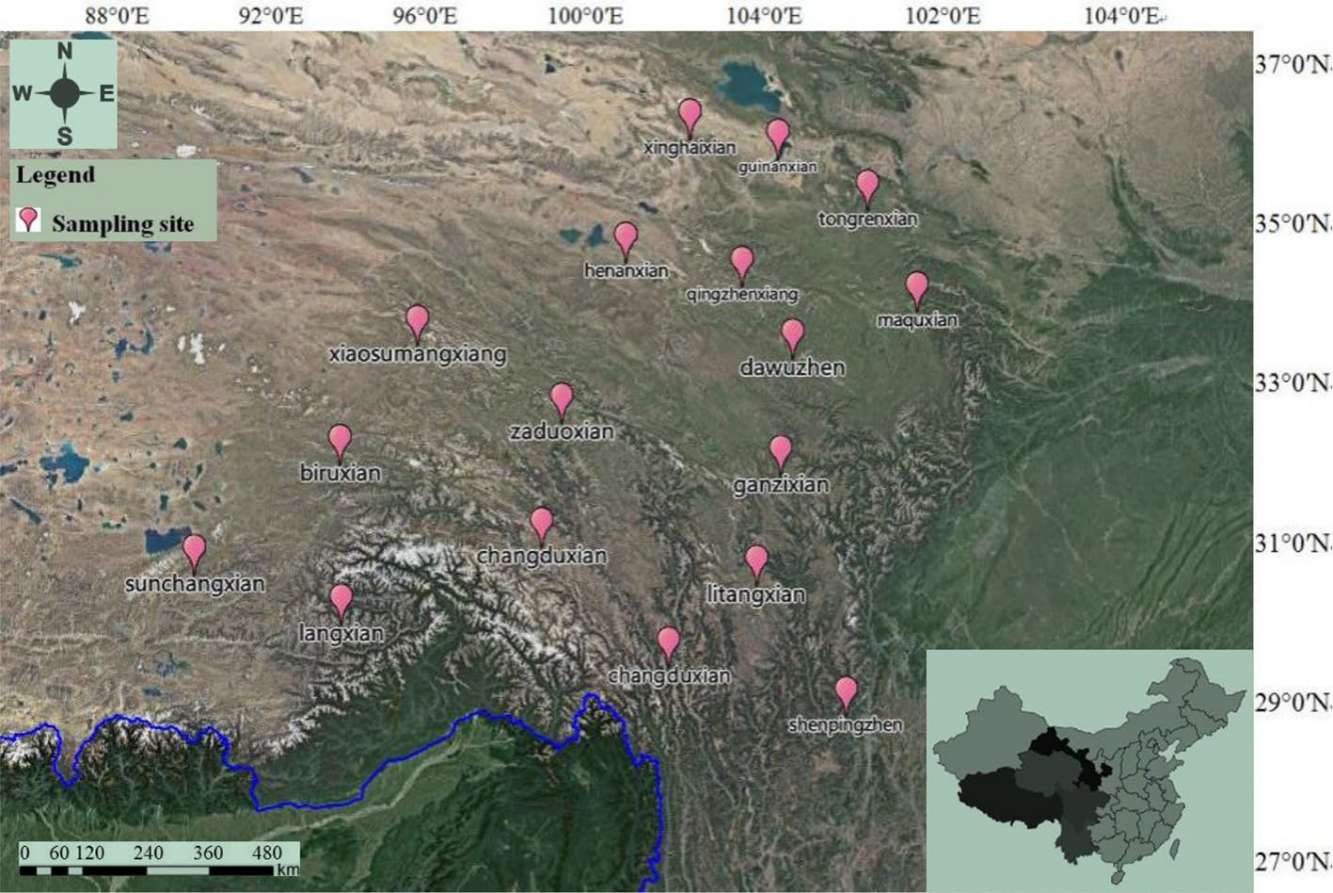
Distribution of sampling sites in China.

Seventeen batches of wild *C. sinensis* were collected in this study. These samples came from some production areas in China—Qinghai, Tibet, Gansu, Yunnan, Sichuan, and other regions. The production area, latitude, and other information of the sample are shown in **Figure 1** and **Supplementary Table 1**. All the residual soil in the sample was carefully washed with ultrapure water. After drying at room temperature, the sample was pulverized and passed through a sieve of 0.3 mm to obtain powder. All samples were authenticated by Mr. Shuai Kang, who was an associate researcher on the identification of medicinal materials in National Institutes for Food and Drug Control (NIFDC). All the samples have stored in traditional Chinese drugs museum and where it belongs to NIFDC.

### Reagents and Materials

Nitric acid (HNO_3_, 65.0%) was of ultrapure quality (Merck, Munchen, Germany). Analytical-grade ammonium carbonate ([NH_4_]_2_CO_3_), sodium hydroxide (NaOH), sodium chloride (NaCl), ethanol (C_2_H_5_OH), acetone (CH_3_COCH_3_), n-hexane (C_6_H_14_), chloroform (CHCl_3_), and hydrochloric acid (HCl) were all purchased from Beijing Chemical Reagent Co. (Beijing, China). Tris was purchased from Roche Diagnostics Gmbh (Berlin, Germany). Purified pepsin was purchased from Sigma-Aldrich (Sigma-Aldrich, USA). Deionized water (18.2 MΩ) was produced using a Millipore ultrapure water system (Millipore, Bedford, USA). The total arsenic standard solution (100.0 µg ml^-1^), dimethyl arsenic (DMA), monomethyl arsenic (MMA), arsenate [As(V)], arsenobetaine (AsB), and arsenocholine (AsC) standard solution were purchased from National Standard Material Research Center (Beijing, China). The arsenate [As(III)] standard solution was purchased from the National Institute of Standards and Technology (Gaithersburg, USA). Standard working solutions of AsB, As(III), DMA, AsC, MMA, and As(V) were prepared by diluting stock solutions immediately before use. The internal standard solution containing germanium (m/z = 74, 100.0 µg ml^-1^) was purchased from Agilent (Agilent Technologies, Folsom, CA, USA).

### Total Arsenic Determination by ICP-MS

For total arsenic analysis, all samples were digested using a MARS 5 microwave digestion system (CEM, USA). The digestion method listed in the Pharmacopoeia of the People’s Republic of China (Chinese Pharmacopoeia Committee, 2015). Approximately 0.2 g of the sample was weighed into a PTFE digestion tube, and then 8 ml of HNO_3_ was added in sequence. The microwave digestion program was as follows: heating for 3 min to 120°C and holding for 3 min, heating for 2 min to 150°C and holding for 3 min, heating for 2 min to 200°C and holding for 12 min. After removing the excess nitric acid, the digested solutions were allowed to cool to room temperature. The solution was then transferred to a polyethene flask and diluted with deionized water to 50 ml. All samples were filtrated through hydrophilic microporous membrane filters (0.45 µm, Nylon 66, Jinteng, China) before determination by ICP-MS (Agilent 7700X, Agilent Technologies Co., USA). Standard working solutions of total arsenic (0–50 µg L^-1^) were prepared by diluting of stock solutions immediately before use. Take germanium as internal standard, and simultaneously enter the sample and internal standard.

### Arsenic Speciation Analysis by HPLC-ICP-MS and SEC-HPLC-ICP-MS

Speciation analysis of the arsenic species in the extracts was conducted by directly coupling high-performance liquid chromatography (HPLC, Agilent 1200 HPLC Pump, USA) with ICP-MS. The separation of six arsenic species, namely, AsB, As(III), DMA, AsC, MMA, and As(V), was performed using an anion exchange column (DioncxIon Pac™ AS7 Analytical column, 250×4.6 mm, USA) with a gradient mobile phase of [NH_4_]_2_CO_3_. In this study, we used a previously validated test method (Zuo et al., 2018). The gradient elution using [NH_4_]_2_CO_3_ and water solutions A (100 mmol L^-1^) and B (deionized water) was carried out (Zuo et al., 2018). The step-gradient program was as follows: 10% A linearly increasing to 50% A from 0 to 3 min, linearly increasing to 100% A from 3.0 to 4.0 min, remaining at 100% A from 4.0 to 11 min, linearly reduce to 10% A from 11 to 13 min, re-equilibration to the initial concentration of 10% A and 90% B from 13 to 17 min and remaining until completion of the separation run. The flow rate of the mobile phase was set to 0.5 ml min^-1^, and the injection volume was 10 µl. The size exclusion chromatography (SEC) condition was performed using a gel column (Shodex PROTEIN KW-802.5, Japan) with an Isocratic mobile phase of Tris-HCl (30 mmol L^-1^, pH = 7.5, prepare 30 mmol L^-1^ Tris first, then adjust the pH to 7.5 with hydrochloric acid), the flow rate of the mobile phase was set to 1.0 ml min^-1^, and the injection volume was 20 µl. The analysis time of SEC-HPLC-ICP-MS was 20 min.

The ICP-MS instrument was tuned and optimized for *m/z* 75 at the beginning of the experiment every day. A standard torch was used with a plasma gas flow rate of 15 L min^-1^, a carrier gas flow rate of 1.0 L min^-1^ and a makeup gas flow rate of 0.25 L min^-1^. The plasma RF power was 1,550 W in the experiment. The signal at m/z 75 for arsenic was monitored and collected in the time-resolved analysis mode (TRA). The integration time for arsenic at m/z 75 was 0.3 s. The six stock solutions were prepared by appropriate dilutions of corresponding standards in doubly deionized water. Working solutions (0–500 µg L^-1^) for arsenic species were prepared daily by diluting the stock solution (1,000 µg L^-1^). All solutions were stored at 4°C until analysis.

### Extraction of Bioaccessible Arsenic

0.25 g of each sample powder was accurately weighed and placed into a 50 ml centrifuge tube, followed by the addition of 10 ml of simulated gastric juice. The mixture was extracted by shaking in a water bath at 37°C for 12 h. After cooling, the extract was filtered through hydrophilic microporous membrane filters (0.45 µm) and subsequently analyzed. Briefly, the simulated gastric juice was prepared using 10 g of purified pepsin (Sigma-Aldrich, USA) and 16.4 ml of diluted nitric acid (take 10.5 ml of concentrated nitric acid diluted to 100 ml with water) diluted to 1,000 ml with deionized water.

### Exploring the Trend of Arsenic Transformation

After weighing accurately 0.3 g of the sample powder, 10 ml of 0, 1, 2, 3, 5, 8, 10, 13, and 15% (v/v) nitric acid solution was added. The mixture was extracted by a microwave rapid extraction system (EXPLORER, CEM, USA) at 70°C for 10 min. After cooling to room temperature, all samples were filtered through 0.45 µm membrane filters before determination. A blank solution was prepared using the same method.

### Investigate the Different Distribution of Arsenic in *C. Sinensis*

#### Extraction of Arsenic From Different Proteins

In this experiment, water-soluble protein, salt-soluble protein, alkali-soluble protein, and alcohol-soluble protein in *C. sinensis* were extracted. First, 0.3 g samples were accurately weighed, followed by extraction in 10 ml of water, 5% NaCl, 0.08 mol L^-1^of NaOH and 70% ethanol solution, respectively. After 12 h at room temperature, ultrasonic extraction was performed three times for 20 min each time. The sample was then centrifuged at 5,000 r min^-1^ for 10 min, and the supernatant was combined. Next, ice-cold acetone was slowly added to the supernatant to increase the concentration to 80%. The protein was precipitated in a refrigerator at 4°C for 12 h and then was centrifuged at 5,000 r min^-1^ for 15 min to separate the supernatant. Finally, the organic solvent in the four proteins was blown off with nitrogen to obtain the purified protein (Liu et al., 2015). These proteins were dissolved in 5 ml of 30 mmol ml^-1^ Tris-HCl buffer (pH = 7.5) (Liu et al., 2015), and filtered through hydrophilic microporous membrane filters (0.45 µm). Furthermore, the obtained supernatant was concentrated to 5 ml and used for subsequent analysis.

#### Extraction of Arsenic From Crude Water-Soluble Polysaccharides

The polysaccharide was prepared by water extraction and alcohol precipitation. First, it weighed 0.5 g sample and immersed in 10 ml of water for 12 h. The mixture was extracted by microwave extraction at 85°C and filtered. The filter residue was repeatedly extracted three times. All filtrates were concentrated at 80°C to 1/4 of the original volume. Three volumes of 95% ethanol were added to the filtrate for 24 h, which was centrifuged at 3,000 r min^-1^ for 40 min, and the precipitate was dissolved in 5 ml of hot water. The protein was removed by the Sevag method (chloroform: n-butanol = 4:1, v/v) until no precipitation in the organic phase to obtain a water-soluble crude polysaccharide solution. Protein residue was dissolved in 5 ml of 30 mmol ml^-1^ Tris-HCl buffer (pH = 7.5), and filtered through hydrophilic microporous membrane filters (0.45 µm). Furthermore, the obtained supernatant was concentrated to 5 ml and subsequently analyzed.

#### Extraction of Arsenic From Lipids

After accurately weighing 0.3 g of the sample powder, 30 ml of n-hexane was added and followed by microwave extraction three times at room temperature. Next, the sample was centrifuged at 5,000 r min^-1^ for 10 min, and the supernatants were combined and condensed to 10 ml.

### Analysis, Quality Assurance, and Quality Control

The standard reference material citrus leaf was used during the total arsenic measurement by ICP-MS. The tAs concentration in the citrus leaf was 1.2 mg kg^-1^, which agreed well with the certified values (1.1 ± 0.2 mg kg^-1^). For the speciation analysis, the spiked recoveries for different arsenic species were in the range of 85–118%, with an RSD < 10% (n = 6). The six arsenic species showed a good linear relationship, and the acceptance criterion coefficients of linear regression (R^2^) were ≥ 0.9990. These results met the quality requirements for metal analysis.

### Health Risk Assessment

To assess the health risks of arsenic in *C. sinensis*, the risk assessment method used the following formula (USEPA, 1998).

(1) $$\mathrm{EDI}=\frac{C\times \mathrm{IR}}{\mathrm{BW}}$$

In the formula, EDI (µg/BWkg day) is the estimated daily intake of arsenic, C (mg kg^-1^) is the concentration of arsenic, IR is the *C. sinensis* intake for an adult from the Chinese Pharmacopoeia 2015 (g person^-1^ day^-1^) (Chinese Pharmacopoeia Committee, 2015), and the maximum daily dose was 9 g in Chinese Pharmacopoeia, which was used in this equation to provide the “worst-case” scenario. BW (kg) is the average body weight and was considered to be 60 kg in this study (USEPA, 2013).

(2) $$\mathrm{HQ}=\frac{\mathrm{EDI}}{\mathrm{RfD}}\times \frac{\mathrm{EF}\times \mathrm{ED}}{\mathrm{AT}}$$

HQ is the hazard quotient of arsenic. If the HQ value is above 1, toxic risk exists, with an increasing possibility as the value increases. RfD is the oral reference dose (µg kg^−1^ day^−1^), and its value of arsenic is 0.3 µg kg^−1^ day^−1^ (USEPA, 2015). EF is the exposure frequency (365 d year^-1^). ED is the exposure duration (70 years). AT is the average exposure time (25,550 days) (Liu et al., 2013; Liu L et al., 2018).

The cancer risk (CR) associated with iAs exposure for *C. sinensis* consumers was calculated according to the following equation:

(3) $$\mathrm{CR}=\mathrm{EDI}\times \mathrm{SF}\times \frac{\mathrm{EF}\times \mathrm{ED}}{\mathrm{AT}}$$

where SF (BWkg day µg^-1^) is the cancer slope factor set by USEPA only for iAs (USEPA, 2015; Liu L et al., 2018). The SF value for iAs was 1.5×10^-3^ BWkg day µg^-1^.

## Results

### Bioaccessible Arsenic in *Cordyceps Sinensis*

**Figure 2:**
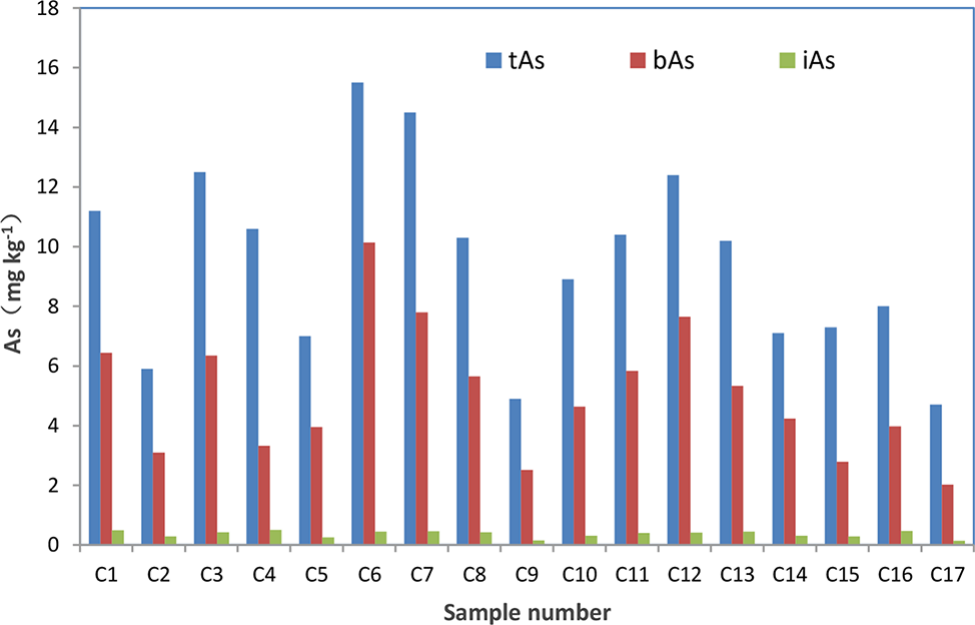
Concentration of tAs, bAs and iAs in *C. sinensis*. The red represents the concentration of bAs. The green represents the concentration of tAs.

**Figure 3:**
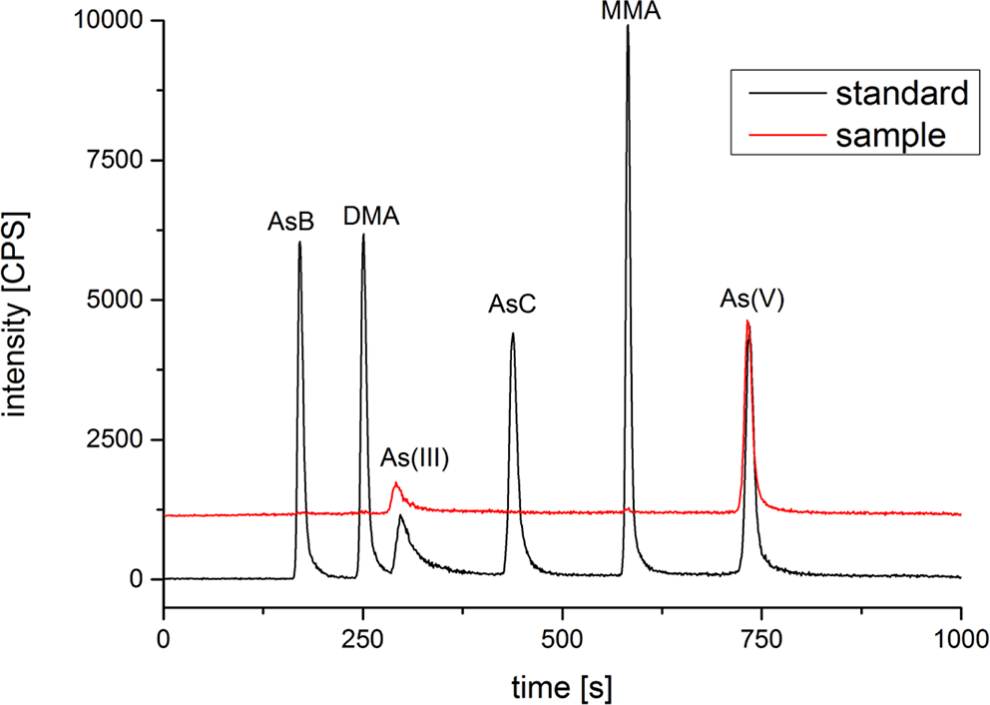
Chromatogram of arsenic species with sample and standards by HPLC-ICP-MS.

**Figure 4:**
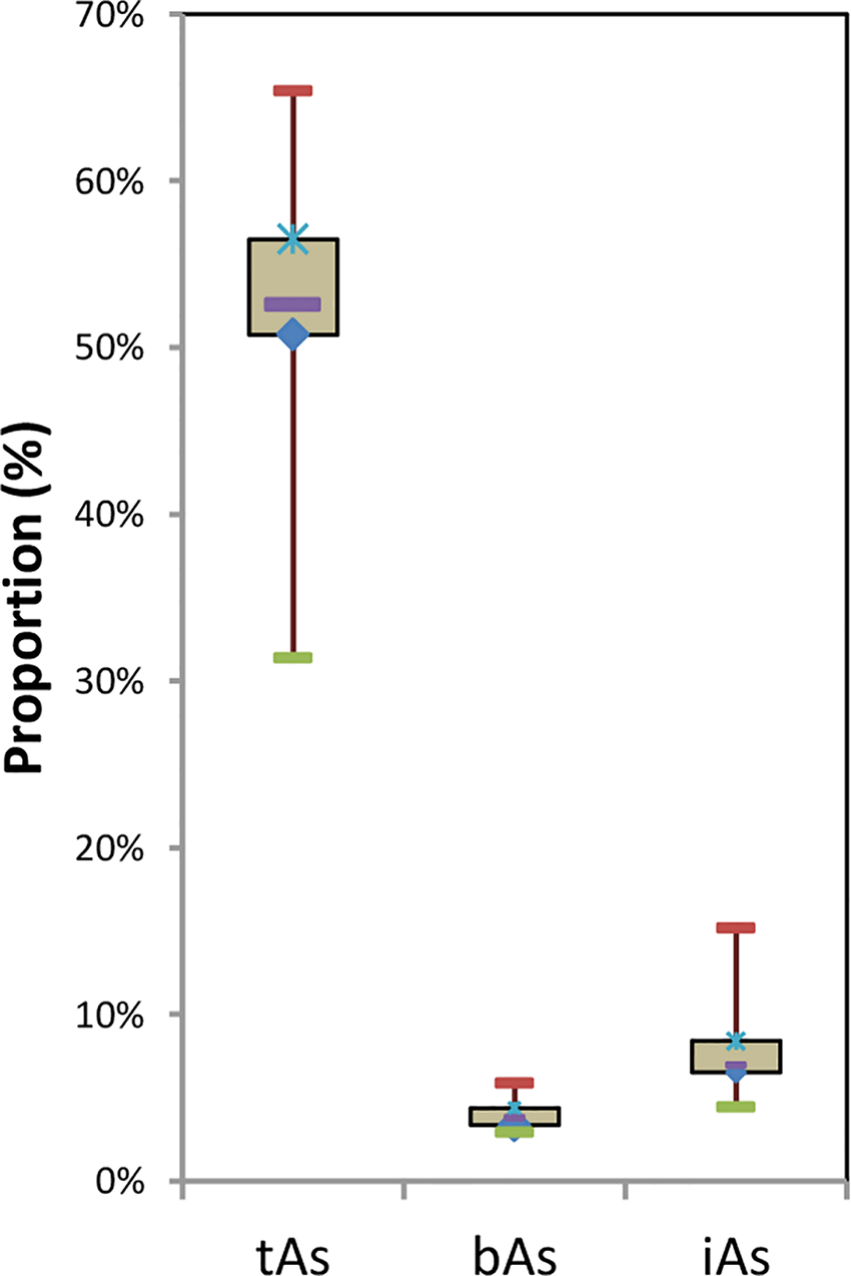
Proportion of tAs, bAs and iAs in *C. sinensis*.

After treated samples by simulated gastric juice, the extracts were detected by HPLC-ICP-MS, and tAs was determined by ICP-MS. According to the measurement results (**Figure 2**), the tAs in 17 batches of *C. sinensis* ranged from 4.7 to 15.5 mg kg^-1^, with an average content of 9.5 mg kg^-1^, which was consistent with some previous studies (Liu et al., 2016; Zhou et al., 2017; Li et al., 2019). The bAs content ranged from 2.0 to 10.1 mg kg^-1^, and the average content was 5.0 mg kg^-1^. There were little As(III) and As(V) detected in this experiment. The typical chromatographic are shown in **Figure 3**. As(III) content ranged from 0.05 to 0.2 mg kg^-1^, and the average content was 0.1 mg kg^-1^. The As(V) content ranged from 0.07 to 0.3 mg kg^-1^, the average content was 0.2 mg kg^-1^, and the total amount of iAs was only 0.4 mg kg^-1^. Further, there were huge differences among tAs, bAs, and iAs (**Figure 4**). The bAs accounted for 52% of tAs, while iAs accounted for 4%. It should be noted that only 8% of free iAs in the bAs was detected by HPLC-ICP-MS. Many chromatographic conditions were tried in this study and did not detect other arsenic species. Thus, its need to further verify whether there is unknown arsenic species present in *C. sinensis*.

### Trend of Arsenic Transformation

**Figure 5:**
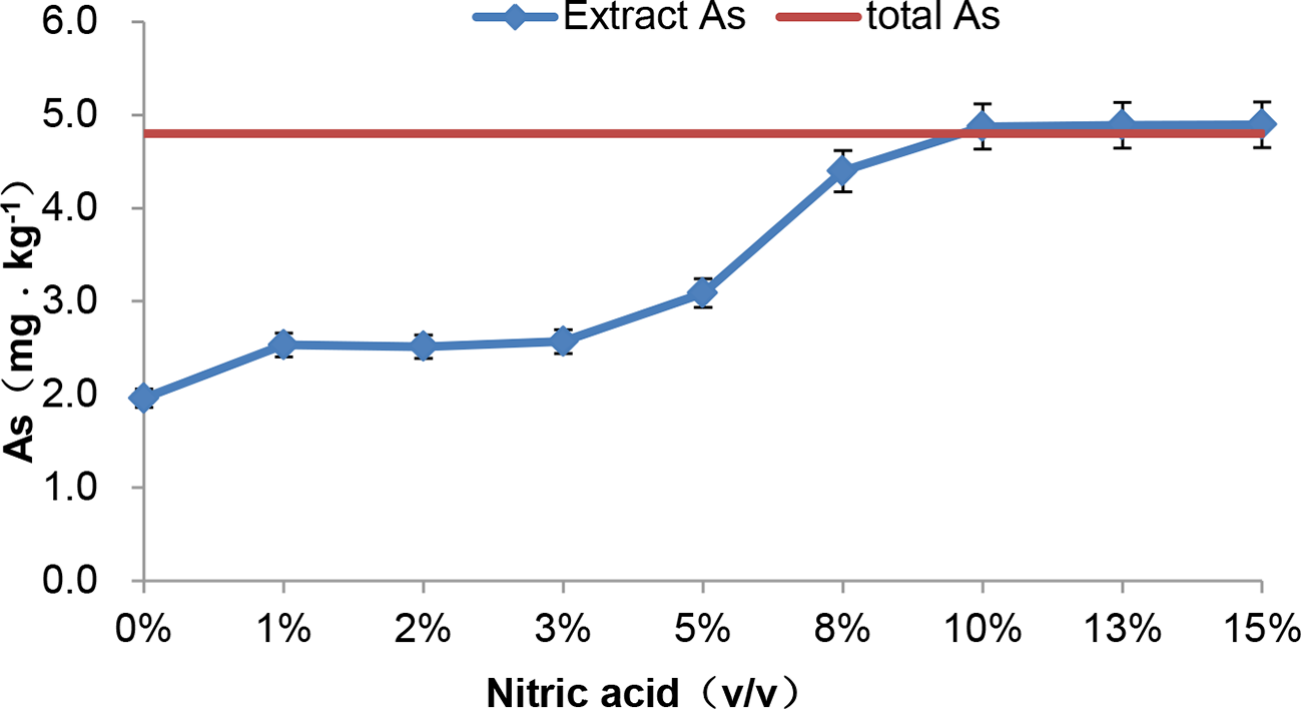
Concentration of arsenic in *C. sinensis* by extracted with different ratios of nitric acid.

In previous study, we found that microwave extraction with nitric acid was effective under the control of the acid concentration and temperature (Zuo et al., 2018). After extraction with nitric acid from 0 to 15% (v/v), tAs and species of arsenic were measured respectively. First, it was showed that (**Figure 5**), extracted arsenic changed slightly with a low ratio acid (1 to 3%) compared with 0% nitric acid. However, when the acid ratio was increased from 5 to 10%, the extracted arsenic content increased sharply. It was worth noting that the extraction rate was close to 100% with 10% nitric acid.

These extracts were also analyzed by HPLC-ICP-MS, and the chromatogram was shown in **Supplementary Figure 1**. A small amount of As(III) and As(V) extracted with low-ratio nitric acid (0 to 3%) was detected, however, the As(III) and As(V) content gradually increased with the acid concentration. Thus, we speculate that unknown arsenic does exist in *C. sinensis*.

### Different Distribution of Arsenic in *Cordyceps Sinensis*

#### Arsenic in Different Proteins, Crude Water-Soluble Polysaccharides and Lipids

**Figure 6:**
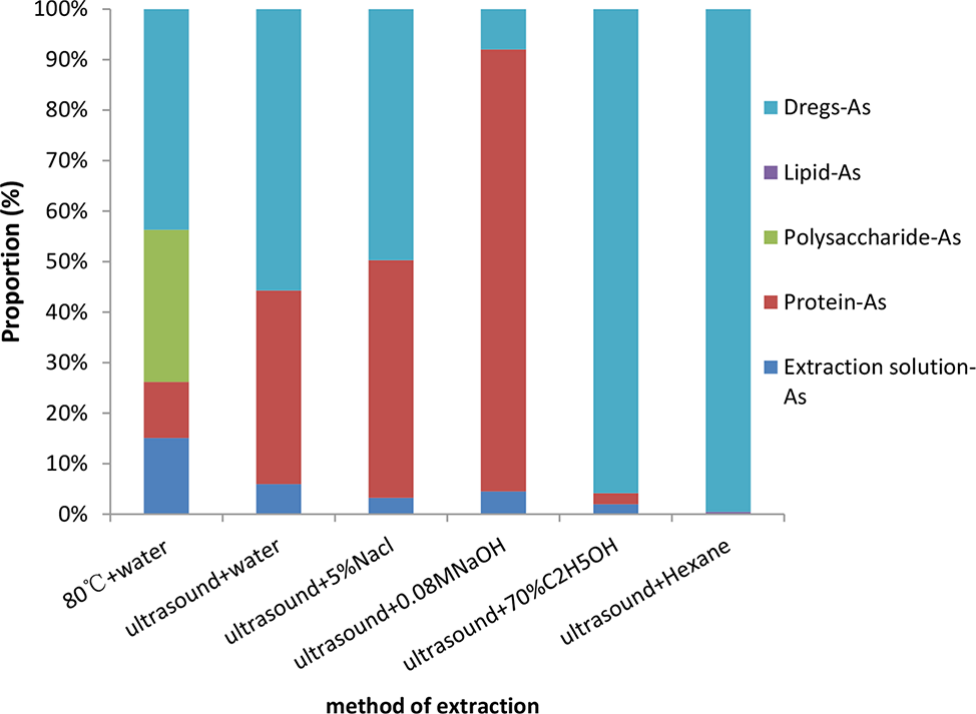
Different distribution of arsenic in *C. sinensis*.

Based on the above research, we speculate that unknown arsenic may be combined with macromolecules such as proteins, polysaccharides, and lipids. We extracted proteins, polysaccharides, and lipids using established methods. Meanwhile, arsenic was measured from these materials (both measured in the extracts and dregs). It should be noted that, since the organic solvent causes the ICP-MS signal drift and lose its authenticity, it needs to evaporate out before measurement. The results of arsenic determination from different materials are shown in **Figure 6**. Arsenic in the crude polysaccharide accounts for 34% of tAs. Compared with different extracted proteins, we found that arsenic in the alkali soluble protein accounted for 82%, the arsenic in water-soluble protein, salt-soluble protein and alcohol-soluble protein were 38, 48, and 2%, respectively. Arsenic in the fat-soluble substance was only 0.4%, and most of the arsenic was not extracted in the dregs.

### Unknown Arsenic Speciation in *Cordyceps sinensis*

**Figure 7:**
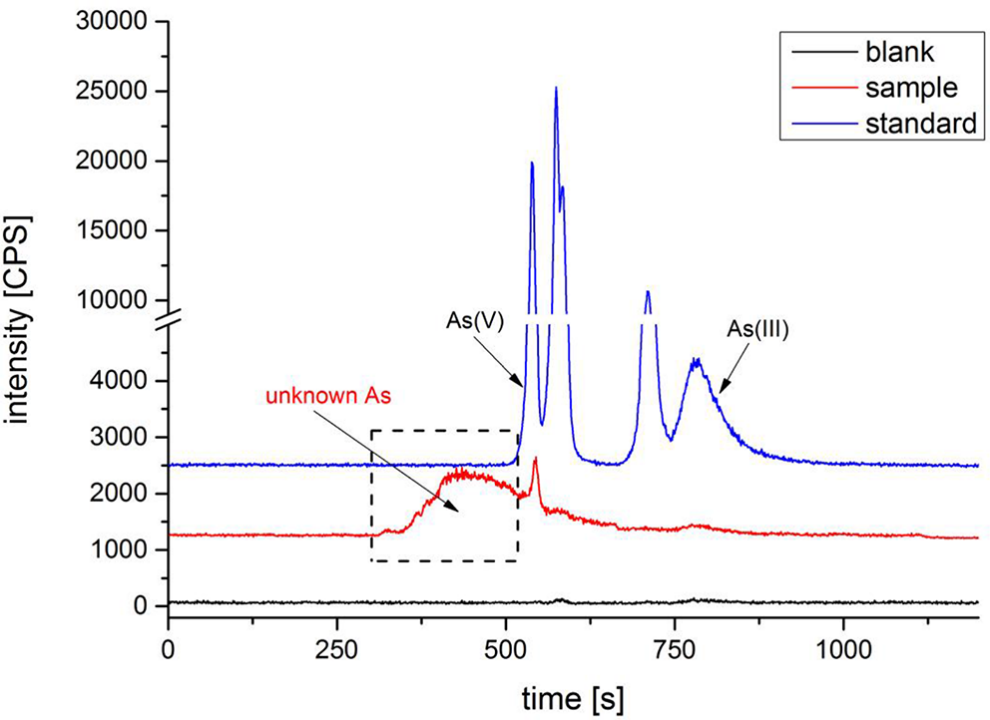
Chromatogram of arsenic species by SEC-HPLC-ICP-MS.

Many attempts have been made, such as anion and cation chromatography, forward and reversed-phase chromatography. No arsenic was detected except for trace amounts of As(III) and As(V). Based on the above results, unknown arsenic in *C. sinensis* may be bind with protein; we established size exclusion chromatography conditions (SEC-HPLC-ICP-MS) to analyze unknown arsenic species. A Shodex PROTEIN KW-802.5 column was used with 30-mM Tris-HCl (pH = 7.5) as the mobile phase (Liu et al., 2015). First, the reference substance, artificial gastric juice extracts and other extracts were analyzed. The chromatogram shows that inorganic arsenics (including As(III) and As(V)) of six arsenic reference substances were well separated and organic arsenic did not separate (**Figure 7**). It was founded that a large unknown peak in sample was eluted before the known arsenic reference; it was thought to be unknown arsenic in *C. sinensis*. After subsequent analysis of different concentrations of nitric acid extracts (**Supplementary Figure 2**), we found that the uAs peak increased gradually with acidity. However, when the acidity exceeds 5%, the large unknown peak disappeared, and As(III) increased gradually (**Supplementary Figure 3**). The uAs conversion trend is consistent with the prediction experiment of this study.

### Health Risk Assessment

The risk assessment of arsenic in *C. sinensis* was carried out according to the formula. According to the 2015 edition of the Chinese Pharmacopoeia, the daily dose of *C. sinensis* is 3 to 9 g (Harris et al., 2011; Chinese Pharmacopoeia Committee, 2015), taking the maximum dose of 9 g per day was used in this study to provide the ‘worst-case’ scenario. According to formulas (1), (2), and (3), we assessed the risk of tAs, bAs, and iAs in *C. sinensis* and expressed them by EDI, HQ and CR.

**Figure 8:**
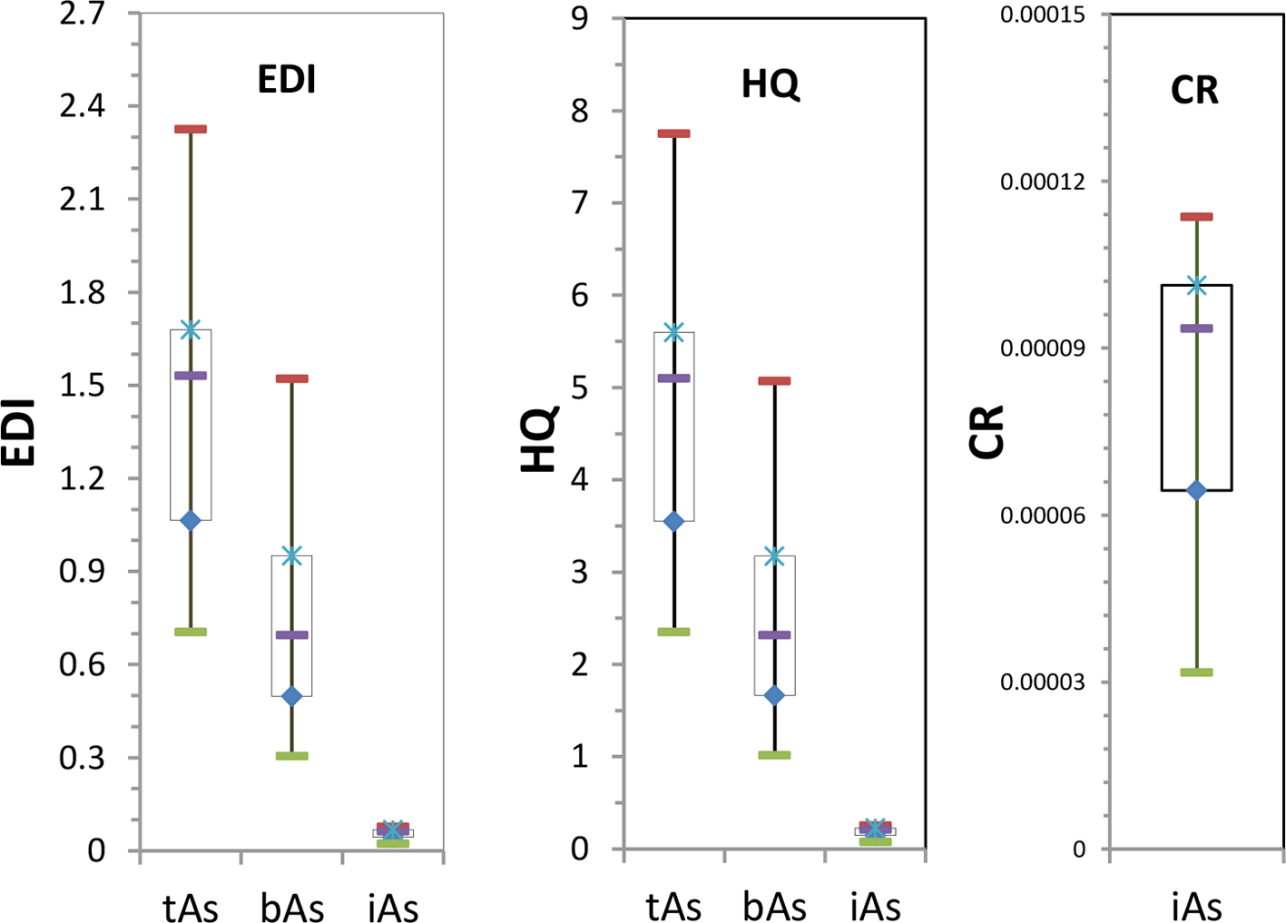
Health risk assessment of arsenic in *C. sinensis*.

From the results (**Figure 8A**), the EDI of tAs in 17 batches of *C. sinensis* was between 0.71 and 2.33 µg kg^-1^ d^-1^, and the average was 1.42 µg kg^-1^ d^-1^. It exceeded 0.3 µg kg^-1^ d^-1^ as specified by USEPA. The EDI of bAs was between 0.30 and 1.52 µg kg^-1^ d^-1^, with an average of 0.76 µg kg^-1^ d^-1^. One batch of EDI was 0.3 µg kg^-1^ d^-1^, while the other EDI exceeded the USEPA regulation. The iAs EDI was 0.02–0.08 µg kg^-1^ d^-1^, and the average was 0.05 µg kg^-1^ d^-1^.

Based on the health risk outcomes (**Figure 8B**), the HQ was between 2.35 and 7.75 with an average of 4.75. Thus long-term use of *C. sinensis* has health risk. The HQ of bAs was between 1.01 and 5.07 with an average of 2.52, HQ of 3 batches was nearly equal to 1, and HQ of the remaining was greater than 1. HQ of the iAs was between 0.07 and 0.25, indicating no risk.

According to **Figure 8C**, the CR value of iAs was between 3.17×10^-5^ and 0.00011, the average value was 0.000082, and the CR value of iAs was within the specified range of 1×10^-6^ - 1×10^-4^ (Ma et al., 2016), indicating no risk.

## Discussion

### Predicting the Binding State of Unknown Arsenic

**Figure 9:**
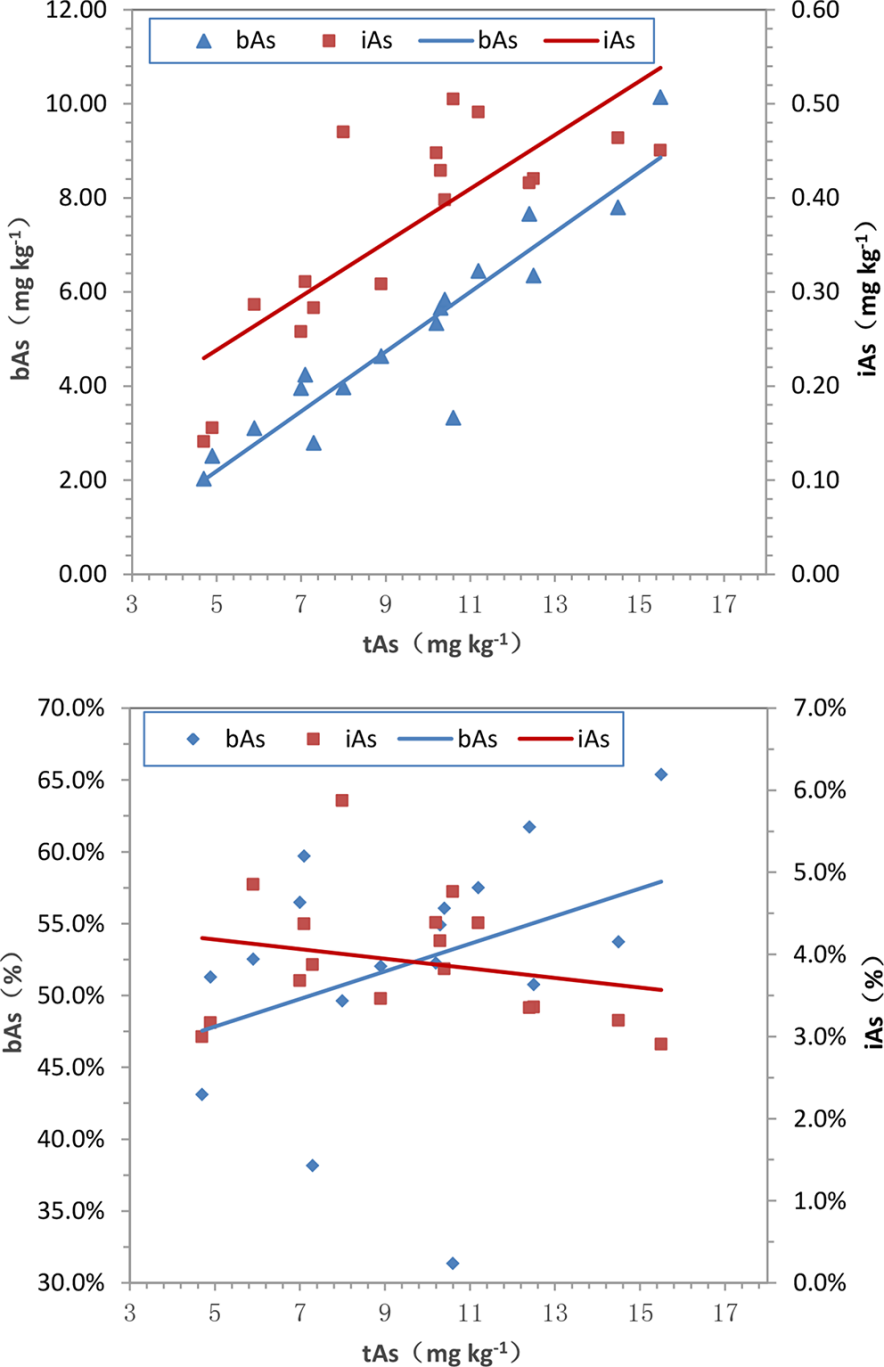
Correlations among tAs, bAs and iAs in *C. sinensis*. (a) tAs-bAs and tAs-iAs, (b) tAs- bAs% and tAs-iAs%. (a-bAs: y = 0.635x-0.984, r = 0.93, p < 0.01; a-iAs: y = 0.029x+0.095, r = 0.80, p < 0.01; b-bAs: y = 0.010x+0.430, r = 0.36, p < 0.01; b-iAs: y = -0.001x+0.045, r = -0.23, p < 0.05.)

The results of the arsenic concentration and bioaccessibility of 17 batches of *C. sinensis* exhibited significant differences. High content of tAs existed in *C. sinensis*, the proportion of bAs was more than 50%, and free iAs was less than 10%. The relationships of tAs-bAs and tAs-iAs were determined by Pearson’s correlation analysis. A positive correlation (p< 0.01) was founded between bAs and tAs (r = 0.93) as shown in **Figure 9A**, indicating the content of bAs was proportional to that of tAs. Similarly, iAs and tAs showed the same trend (r = 0.80, p < 0.01). In addition to the concentration, the percentages of bAs and iAs were used to investigate the correlation with tAs (**Figure 9B**). The percentage of bAs increased as tAs increased (r = 0.36, p < 0.01). Conversely, the percentage of iAs was inversely proportional to tAs (negative correlation, r = -0.23, p < 0.05), this may due to binding states of uAs leads to limited iAs extraction in *C. sinensis*.

Trace amounts of iAs in bAs was detected, and most of the them was unknown. The different distribution of arsenic in *C. sinensis* showed that most of the uAs existed as arsenic-binding protein. As the acidity of the extraction increased, it continuously released into iAs. Wild *C. sinensis* contains 31.73% crude protein (Leung et al., 2006), the amino acids mainly includes glutamic acid, aspartic acid, leucine, and alanine (Leung et al., 2006). Studies have shown that the sulfhydryl group of the protein (i.e., cysteine) is the major binding site for As(III), while the intermediate metabolites of arsenic such as MMA^III^ and DMA^III^ have high affinity with the cysteine residues of proteins and can be combined with various proteins. These proteins including hemoglobin, metallothionein, thioredoxin peroxidase, glutathione reductase, and glycopeptide peroxidase (Mizumura et al., 2010; Shen et al., 2013; Chen et al., 2015; Liu Q et al., 2018). Metallothionein, a metal-binding protein having cysteine residues and high metal content, was founded in living organisms (Ziller et al., 2017; Scheller et al., 2018). Therefore, we speculate that uAs could specifically bind to proteins in *C. sinensis*.

### Discussion on the Health Risks of Unknown Arsenic

From the results of risk assessment, tAs and bAs were founded much more poisoning than free iAs due to high contents. The iAs was the most toxic among the arsenic species. The risk of arsenic in *C. sinensis* cannot be ignored for four reasons: (1) the bAs is 52% and the HQ of bAs is greater than 1, indicating risk. We also treated bAs in simulated gastric juice then added different nitric acids, found that uAs was converted into iAs easily (**Supplementary Figure 4**). Meanwhile, the SEC-HPLC-ICP-MS results showed the same trend (**Supplementary Figure 5**). (2) In this study, the conversion of six known arsenic compounds was also investigated. Each arsenic compound was treated according to the experimental conditions (microwave extraction at 70°C for 10 min with 10% nitric acid), and the recovery and conversion of iAs were determined. According to the results (**Supplementary Figure 6**), large amounts of MMA conversion occurred in oAs, but it was not converted into iAs. Little iAs was found in oAs (**Supplementary Figure 7**), but almost no conversion occurred. This means that almost no oAs was converted into iAs under the experimental condition. It is consistent with previous study (Wei et al., 2019). The result further showed that uAs may bind to proteins in the form of iAs. (3) According to the study, oAs was mainly metabolized into other forms instead of iAs in human body (Navarro Serrano et al., 2016; De Loma et al., 2019). Apart from this, oAs are nontoxic or less toxic because it is difficult to transform into highly toxic iAs (Stice et al., 2016; Taylor et al., 2017). If uAs is released as free iAs in the human body, it may accumulate and causing an inevitable health risk for long-term exposure. (4) In the human stomach, proteins were hydrolyzed by pepsin (Chen et al., 2015); after further destruction of the structure of most proteins, it formed free amino acids and peptides, increased the possibility of arsenic binding to the protein (Shen et al., 2013). In summary, combined with the risk assessment results, the health risks cannot be ignored.

### Recommendations for the Use of Cordyceps and Its Arsenic Regulation

Arsenic accumulation from soil in *C. sinensis* may occur with uptake and metabolism of various elements (Zhu et al., 2017). Research indicated that arsenic content in soil varies in different regions (Ma et al., 2016a). According to our previous study, the accumulation of arsenic in *C. sinensis* is caused by high levels of arsenic in the soil in main producing areas (Li et al., 2019), and the arsenic content in the soil range from 13.61 to 104.07 mg kg^-1^. Conversely, some researchers compared artificially cultivated *C. sinensis* and found that the arsenic content is very low (Liu et al., 2016).

There are two factors of arsenic that affect human health: exposure time and frequency. Presently, it is difficult to solve the problem of the high background value of arsenic in *C. sinensis*. We can only reduce the risk by using frequency and usage. Hence, based on the bAs, we recommend two suggestions for use *C. sinensis*: (1) If the exposure time is for a lifetime, maximum usage should not exceed 4 g per day, (2) If usage is kept the same, the time of continuous use of *C. sinensis* should not extend beyond 5 months per year. According to these two suggestions, the HQ average value is less than 1, and the health risk is acceptable. Therefore, these recommendations can be used as a reference for doctors’ clinical medication guidance.

Presently, no limits on arsenic in *C. sinensis* were specified in the 2015 edition of the Chinese Pharmacopoeia neither in industry standards. The maximum amount of residues should be established combined with the results of the risk assessment. If the limit of arsenic is too strict, it will hinder economic development of the production region. Reversely, it will cause the health risks. Its need balance well. Because uAs in *C. sinensis* has not been identified yet, the relatively accurate risk assessment results cannot be given, thus the standard limit of arsenic in *C. sinensis* cannot be formulated. But the usage and dosage can be modified. For instance, patients need to follow the doctor’s advice, information such as the dose and frequency of use must be clear. Furthermore, it should be indicated in the instruction manual that there is a health risk in using this drug for a long time.

## Conclusions

In this study, the species of arsenic in *C. sinensis* and its potential health risk were investigated. Unknown arsenic in *C. sinensis* was discovered by SEC-HPLC-ICP-MS. Furthermore, arsenic was mainly found in alkali-soluble proteins in *C. sinensis*. Unknown arsenic in *C. sinensis* easily converted into free iAs by the trend of arsenic transformation, which enhanced toxicity. This work not only contributed to the study of the state of arsenic in *C. sinensis*, but also the medicinal and regulatory aspects. The confirmation of unknown arsenic is significant for the safety evaluation and risk assessment of wild *C. sinensis*.

## Data Availability Statement

All datasets generated for this study are included in the article/**Supplementary Material**.

## Author Contributions

SM and HJ designed the study. YaL conducted the experiments, analyzed the data, and wrote the manuscript. SM, YuL, XH, and HJ revised the manuscript. All the authors read and approved the final version of the manuscript.

## Funding

This work was financially supported by the 12th 5 Year National significant new drugs creation feature subjects-traditional Chinese medicine quality safety evaluation and risk control technology platform (No.2014ZX09304307-002).

## Conflict of Interest

The authors declare that the research was conducted in the absence of any commercial or financial relationships that could be construed as a potential conflict of interest.

## Abbreviations

*C. sinensis, Cordyceps sinensis*; As, Arsenic; As(III), Arsenite; As(V), Arsenate; DMA, Dimethylarsinic acid; MMA, Methylarsonic acid; AsB, Arsenobetaine; AsC, Arsenocholine; uAs, Unknown Arsenic; oAs, Organic arsenic; iAs, Inorganic arsenic; bAs, Bioaccessible arsenic; tAs, Total arsenic; EDI, Estimated Daily Intake; HQ, Hazard Quotient; CR, Cancer Risk; TCM, Traditional Chinese medicine; SEC-HPLC-ICP-MS, Size Exclusion Chromatography and High Performance Liquid Chromatography Inductively Coupled Plasma Mass Spectrometry.
