# Supplementary material for: Arsenic Species in Cordyceps sinensis and Its Potential Health Risks
Source: Front Pharmacol. 2019 Dec 6;10:1471. doi: 10.3389/fphar.2019.01471 (PMC6910106; doi:10.3389/fphar.2019.01471)
Supplement: Supplementary file 1 [file DataSheet_1.docx]

**SUPPLEMENTARY INFORMATION**


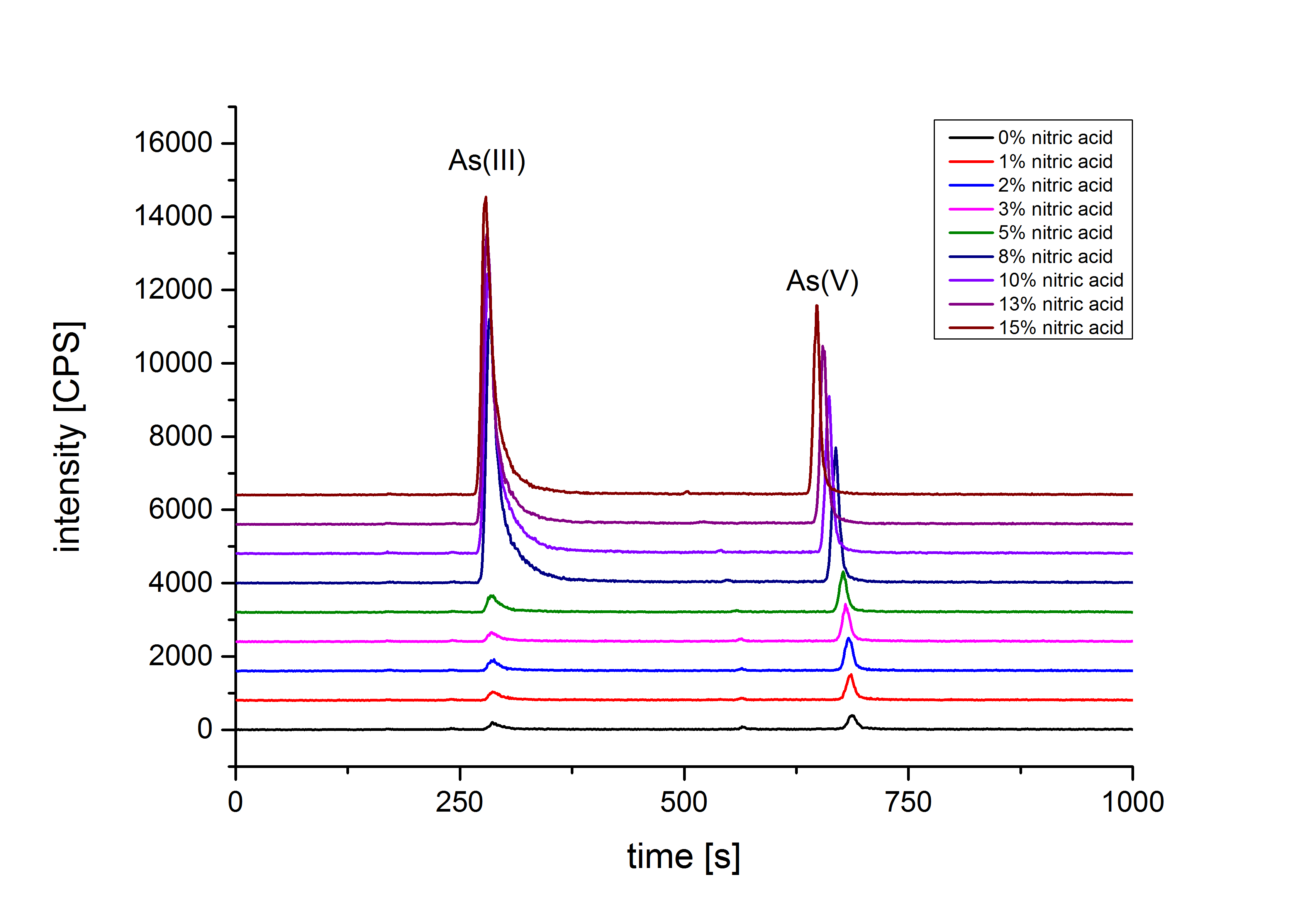


**FIGURE 1.** Chromatogram of arsenic species in *C. sinensis* by extracted with different ratios of nitric acid using HPLC-ICP-MS*.*


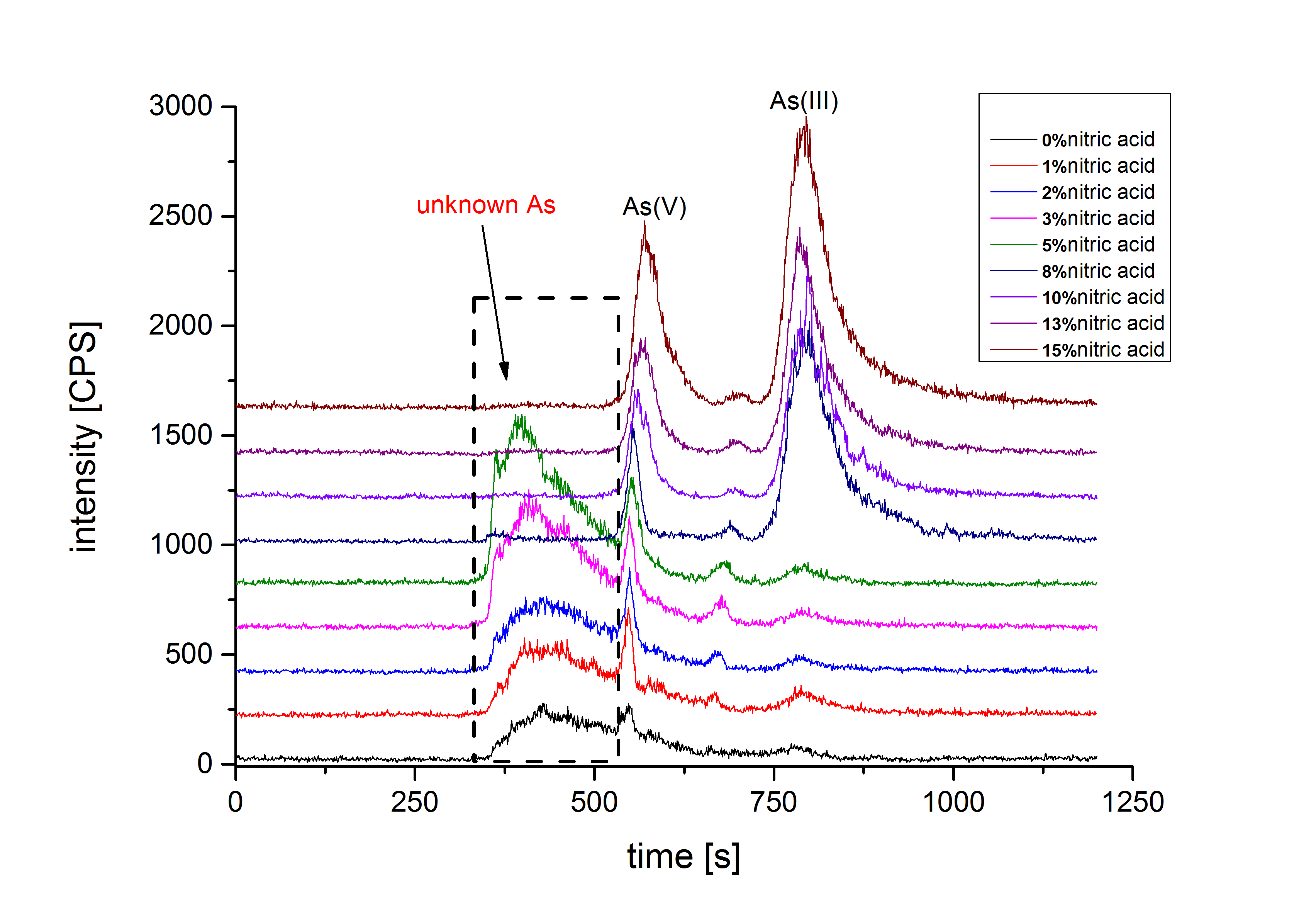


**FIGURE 2.** Chromatogram of arsenic species extracts with different ratios of nitric acid in *C. sinensis* by SEC-HPLC-ICP-MS*.*


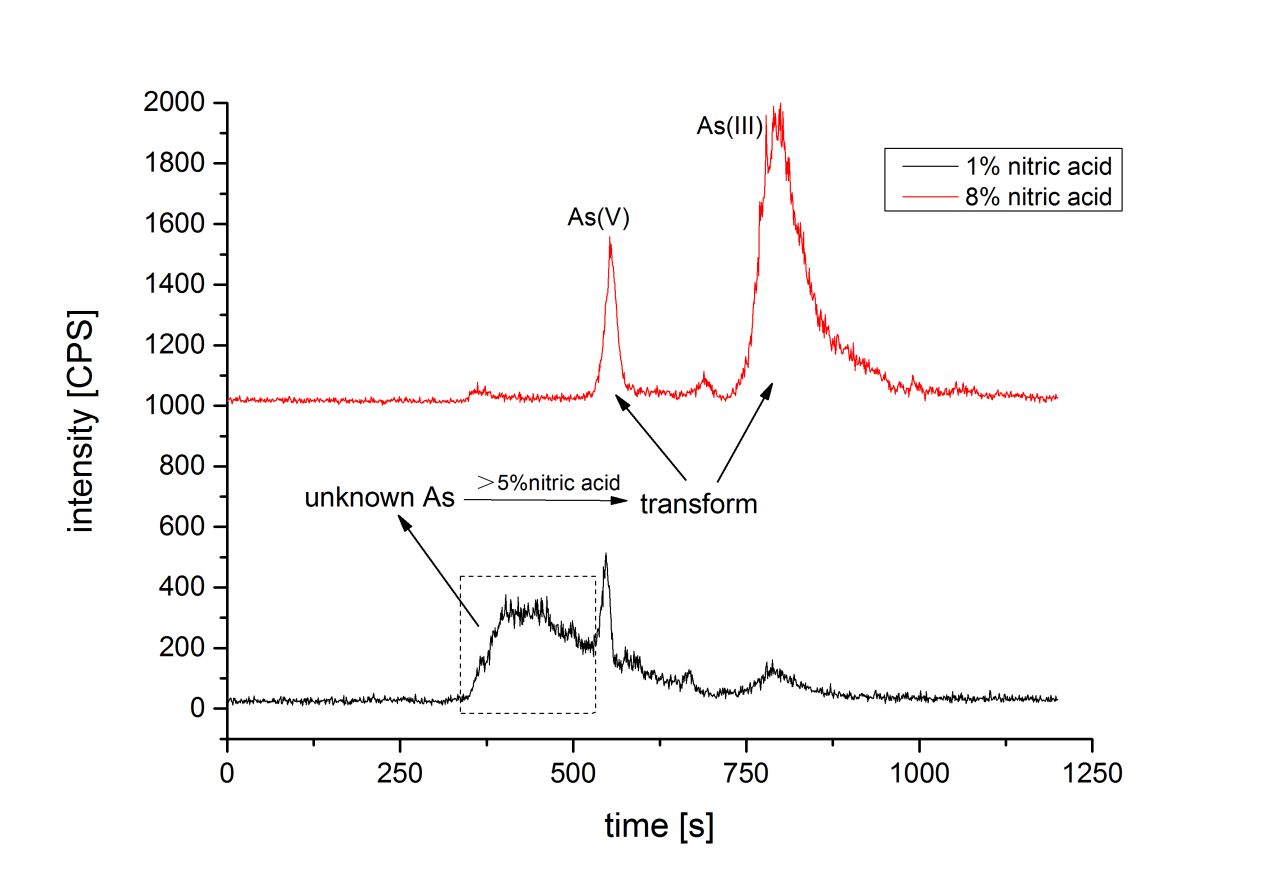


**FIGURE 3.** Chromatogram of unknown arsenic in *C. sinensis* and its conversion behavior in the extracts.


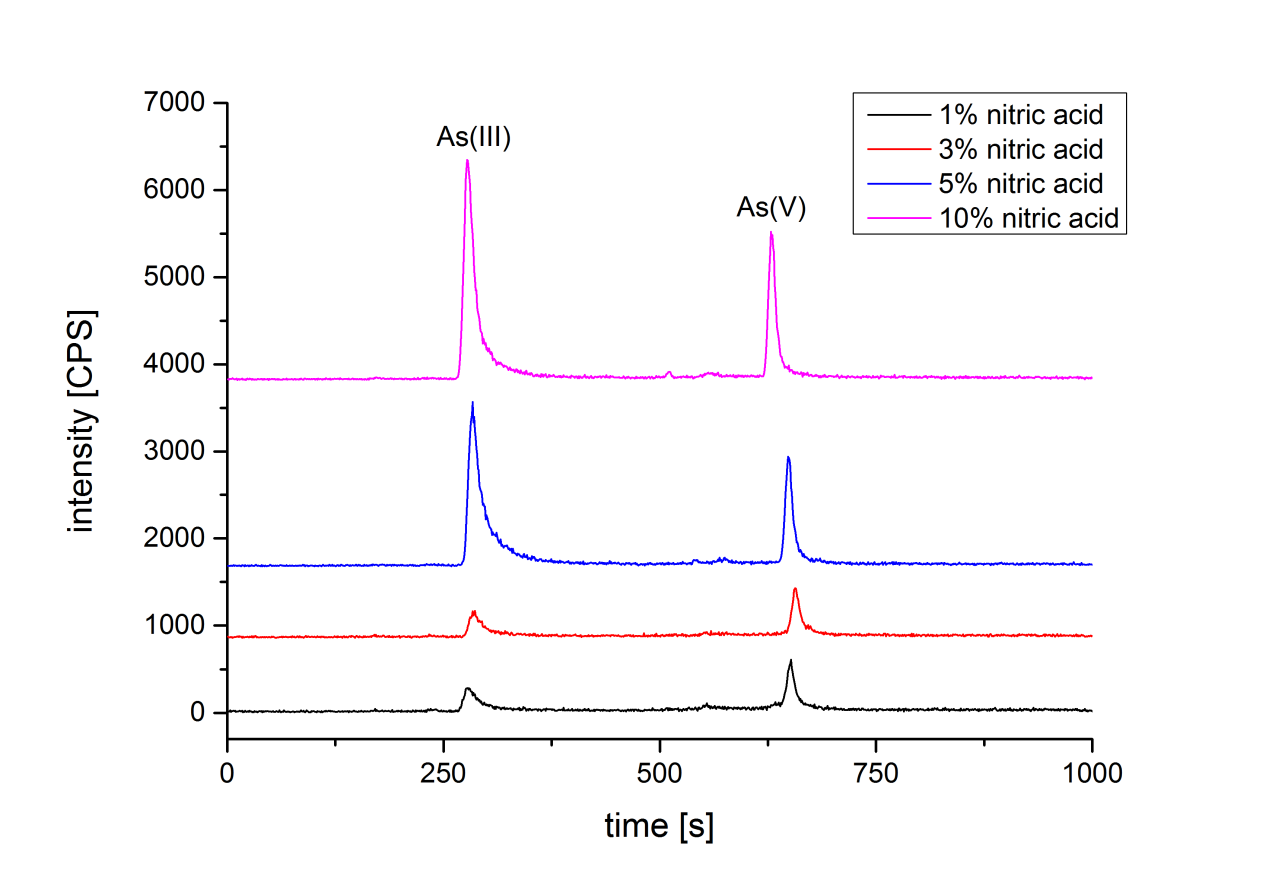


**FIGURE 4.** Chromatograms of simulated gastric juice extracts treated with different proportions of nitric acid in *C. sinensis* by HPLC-ICP-MS.


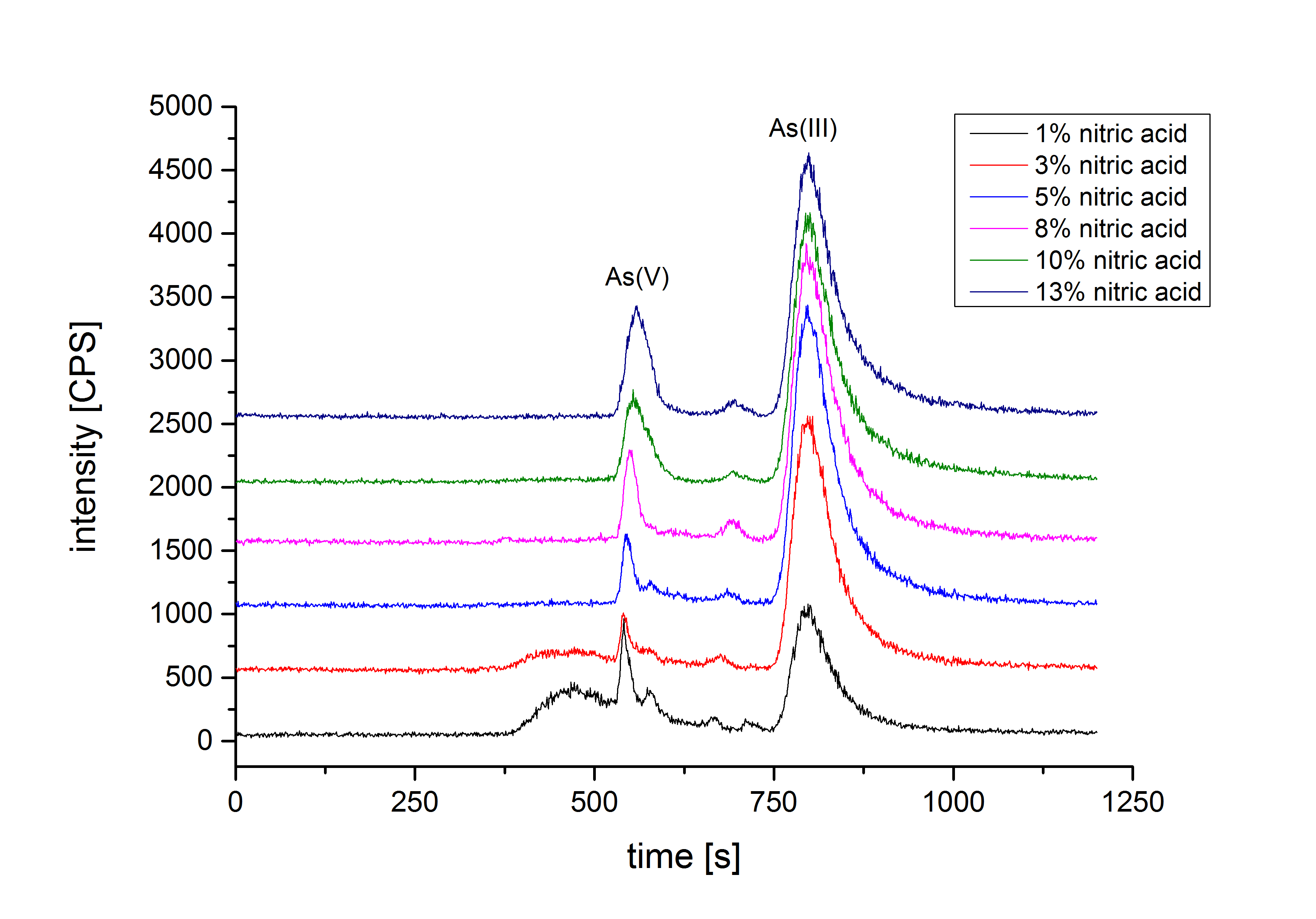


**FIGURE 5.** Chromatograms of simulated gastric juice extracts treated with different proportions of nitric acid in *C. sinensis* by SEC-HPLC-ICP-MS.


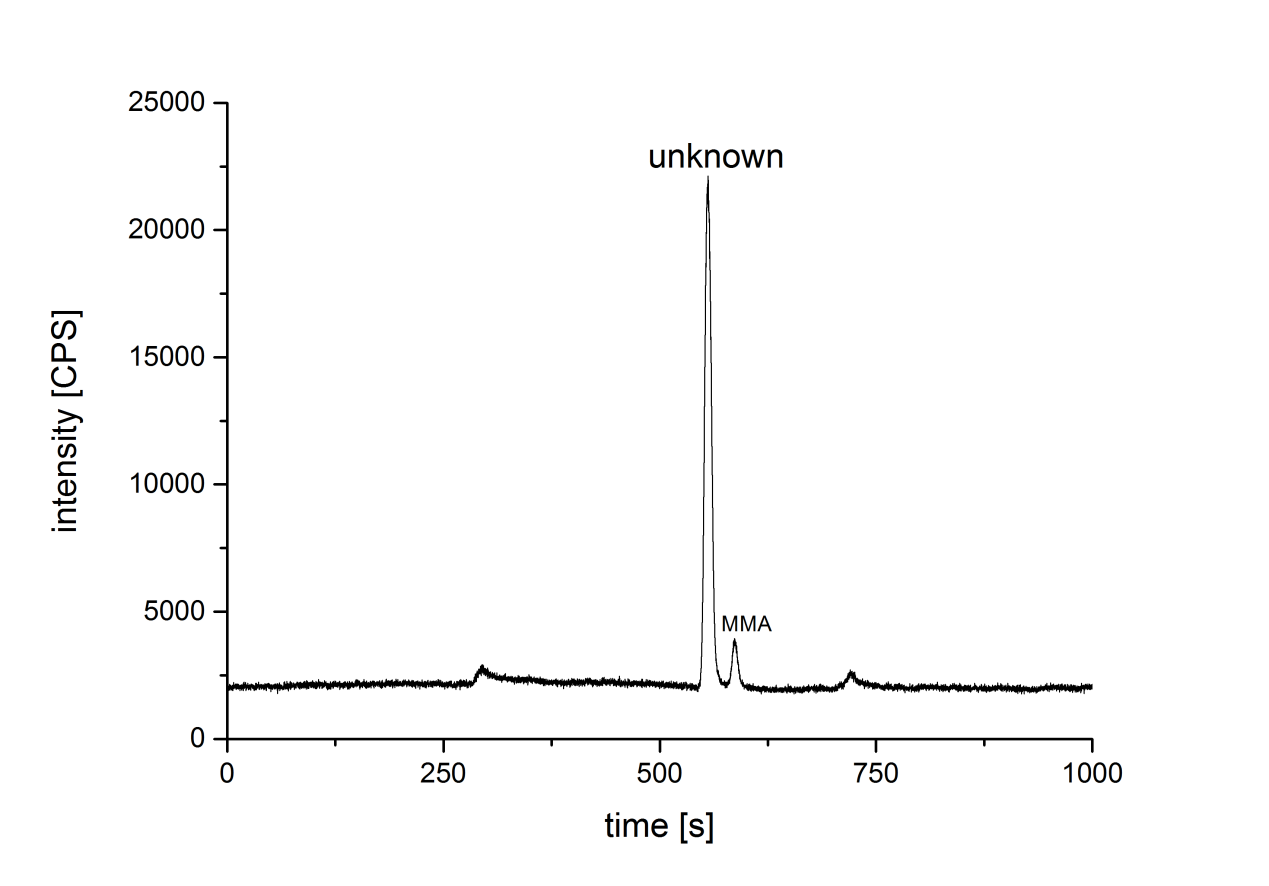


**FIGURE 6.** MMA conversion chromatogram under extracted conditions.

**FIGURE 7.** The results of arsenic transformation .

**TABLE 1.** Sample collection information in present study

| Code | Location | Latitude | Longitude |
| --- | --- | --- | --- |
| C1 | Qinghai | 32°33^′^17N | 097°12^′^17E |
| C2 | Qinghai | 32°56^′^31N | 095°43^′^00E |
| C3 | Qinghai | 34°13^′^22N | 100°14^′^02E |
| C4 | Qinghai | 34°13^′^48N | 100°01^′^50E |
| C5 | Qinghai | 35°26^′^45N | 100°36^′^21E |
| C6 | Qinghai | 36°00^′^40N | 099°44^′^01E |
| C7 | Qinghai | 34°04^′^24N | 102°07^′^08E |
| C8 | Qinghai | 34°04^′^24N | 102°16^′^57E |
| C9 | Tibet | 31°21^′^19N | 097°42^′^22E |
| C10 | Tibet | 31°04^′^25N | 096°58^′^48E |
| C11 | Tibet | 31°48^′^33N | 093°34^′^18E |
| C12 | Tibet | 31°44^′^28N | 092°40^′^24E |
| C13 | Gansu | 34°04^′^24N | 102°07^′^08E |
| C14 | Yunnan | 28°24^′^22N | 098°59^′^07E |
| C15 | Sichuan | 31°35^′^06N | 099°41^′^47E |
| C16 | Sichuan | 30°00^′^40N | 100°18^′^56E |
| C17 | Qinghai | 32°33^′^17N | 097°12^′^17E |
